# Aneuploidy is permissive for hepatocyte-like cell differentiation from human induced pluripotent stem cells

**DOI:** 10.1186/1756-0500-7-437

**Published:** 2014-07-08

**Authors:** Fallon K Noto, Megan R Determan, Jun Cai, Max A Cayo, Sunil K Mallanna, Stephen A Duncan

**Affiliations:** 1 Department of Cell Biology, Neurobiology and Anatomy, The Medical College of Wisconsin, 8701 Watertown Plank Road, Milwaukee, WI 53226, USA

**Keywords:** iPSC, Hepatocyte differentiation, Aneuploidy

## Abstract

**Background:**

The characterization of induced pluripotent stem cells (iPSCs) and embryonic stem cells (ESCs) routinely includes analyses of chromosomal integrity. The belief is that pluripotent stem cells best suited to the generation of differentiated derivatives should display a euploid karyotype; although, this does not appear to have been formally tested. While aneuploidy is commonly associated with cell transformation, several types of somatic cells, including hepatocytes, are frequently aneuploid and variation in chromosomal content does not contribute to a transformed phenotype. This insight has led to the proposal that dynamic changes in the chromosomal environment may be important to establish genetic diversity within the hepatocyte population and such diversity may facilitate an adaptive response by the liver to various insults. Such a positive contribution of aneuploidy to liver function raises the possibility that, in contrast to existing dogma, aneuploid iPSCs may be capable of generating hepatocyte-like cells that display hepatic activities.

**Results:**

We examined whether a human iPSC line that had multiple chromosomal aberrations was competent to differentiate into hepatocytes and found that loss of normal chromosomal content had little impact on the production of hepatocyte-like cells from iPSCs.

**Conclusions:**

iPSCs that harbor an abnormal chromosomal content retain the capacity to generate hepatocyte–like cells with high efficiency.

## Background

The availability of human pluripotent stem cells has provided a cell culture platform for study of human disease and development [1]. Pluripotent cells could also potentially be used therapeutically as a source of cells for transplant or drug discovery. Moreover, the finding that patient–specific pluripotent cells can be relatively easily generated by molecular reprogramming raises the prospect of using personalized regenerative medicine to treat a variety of diseases, arguably without fear of immune rejection [2,3]. While the biomedical potential of pluripotent stem cells is irrefutable, to realize such potential requires an in depth understanding of the fundamental properties and complications that are associated with genomic changes that accompany the reprogramming process. Many studies have revealed that, as a consequence of reprogramming and stem cell culture, genetic instability commonly occurs [4]. The genetic variations that have been observed are diverse and include copy number variations (CNVs), chromosomal rearrangements, and several sub-chromosomal mutations including deletions and point mutations [5-10]. For pluripotent cells to be used safely in regenerative medicine, substantial characterization would therefore be necessary to ensure the genomic integrity of transplantable cells.

Although it is clear that iPSC–derived cells used for cell therapy should be euploid due to the need for safety, how chromosomal variation affects the production of differentiated cells in culture remains ill defined. Cell differentiation is an orchestrated process that relies on complex extracellular signals coupled with epigenetic and genetic responses, which establish transcription factor networks that drive cell fate. With this in mind it would seem intuitive that aneuploid genomes would be detrimental to the differentiation of a specific cell type. However, analyses in both mice and humans have shown that, unlike most somatic cells, the chromosomal content of proliferating hepatocytes is diverse and that aneuploidy is commonly observed [11,12]. In liver damage models it has been established that changes in chromosomal content are dynamic with cells constantly gaining and losing chromosomes. This dynamic nature of chromosomal imbalances found in hepatocytes has been referred to as the 'ploidy conveyer’ and it has been hypothesized that the conveyer may facilitate an adaptive response of hepatocytes to noxious environments [11]. With this model in mind, it is formally possible that hepatic function may best be recapitulated if hepatocytes are generated from iPSCs that display genomic variation. The generation of polyploidy in rodent hepatocytes begins after weaning and increases as the animals age; however, the impact of aneuploidy on hepatocyte differentiation is unknown. We, therefore, addressed whether hepatocyte-like cells could be generated from iPSCs that are karyotypically abnormal.

## Results

### Characterization of aneuploid iPSCs

We have previously described the generation of a human iPSC line, referred to as iPSC-K3, which was produced by reprogramming foreskin fibroblasts using transient transfection of plasmids that express OCT4, NANOG, LIN28, and KLF4 [13]. These iPSCs are free of integrated exogenous DNA and have a high capacity to differentiate into cells that display characteristics of hepatocytes. The iPSC-K3 cells maintain a stable karyotype; however, as with all human pluripotent stem cells, colonies spontaneously arise that contain chromosomal variations that presumably support their efficient proliferation in culture [4].

**Figure 1:**
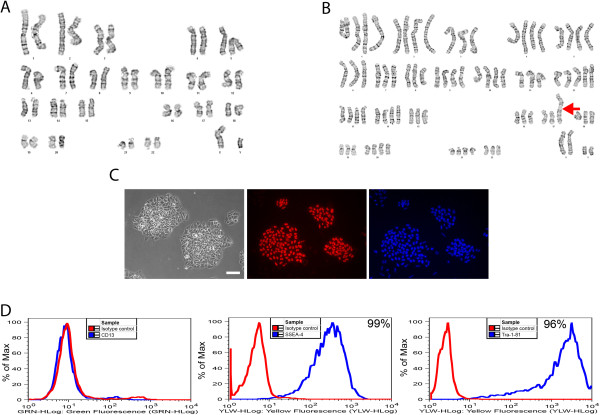
**Characterization of iPS cells with abnormal karyotype. A)** Control iPSC-K3 cells were generated from human foreskin fibroblasts and have a normal diploid male (46, XY) karyotype. **B)** A sub-line of iPSC-K3 cells, referred to as iPSC-K3^aneuploid^, have an abnormal near-tetraploid karyotype as well as rearrangements in chromosome 17 (red arrow). The karyogram presented here is representative of the cells analyzed. **C)** Left panel shows a phase contrast micrograph of iPSC-K3^aneuploid^ cells in culture. Immunocytochemistry revealed the presence of OCT4 (middle panel) and DAPI was used to identify cell nuclei. Scale bar = 100 μm. **D)** FACS analysis reveals that iPSC-K3^aneuploid^ cells do not express a marker of differentiated cells, CD13 (left panel); however, pluripotent markers SSEA-4 (middle panel) and Tra-1-81 (right panel) are expressed on the surface of 99% and 96% of the cells, respectively. Isotype controls for each antibody are indicated by the red line, antibodies by the blue line.

During routine analyses of a subculture of iPSC-K3 cells we identified a cell line that was extensively aneuploid (Figure 1 and Table 1). In this culture, the majority of the cells approached a tetraploid state, but included both gain and loss of discrete chromosomes as well as chromosomal rearrangements. The composite karyotype of the line, determined by examination of 19 cells with consistent abnormalities, is XXYY,-3[19],-6[18],-7[5],-8[18],-11[16],+12[19],-13[3],-14[4],-15[19],-16[12],-17[5],dup(17)(q11.2q25)ins(17)(q25;q25q11.2)[19],-18[12],-19[19],-22[11]. These 19 cells contained 79 ~ 87 chromosomes per cell and the frequency of a given chromosomal imbalance is indicated within the square brackets ([n]). The major chromosomal imbalances observed were a gain of chromosome 12 and loss of chromosomes 3, 6, 7, 8, 11, 13, 14, 15, 16, 17, 18, 19 and 22. In addition, in all 19 cells we observed an unbalanced structural abnormality of chromosome 17 that represented a gain of long (q) sequences from chromosome 17 (dup(17)(q11.2q25)ins(17)(q25;q25q11.2)). Rearrangements of chromosome 17 have been commonly found in both human ESCs and iPSCs and are believed to confer advantageous growth in culture [8]. Although all cells examined were abnormal not all cells contained every observed rearrangement. Chromosomal imbalances found in a minority of cells included loss of chromosomes 7, 13, 14, 18, and 22, raising the possibility that the culture arose from a mixed population. However, given that all cells approached tetraploidy, we favor an alternative explanation in which the cells originated clonally from a single tetraploid event and subsequently diverged in culture. Figure 1 shows karyograms of the parental iPSC-K3 line and a single cell from the aneuploid iPSC-K3 (iPSC-K3^aneuploid^) derivative that contains the majority of the chromosomal abnormalities that were identified and Table 1 describes the frequency of each abnormality. In addition to the majority of the cells that displayed a shared karyotype, a single non-clonal cell was identified with the karyotype 66,XXY,+1,+2,-3,+4,+5,+9 + 12,-14,-15,-16, dup(17)(q11.2q25)ins(17)(q25;q25q11.2),-19,-19,-21,-21,-22.

**Table 1:** **Chromosomal abnormalities identified in of iPSC-K3**^**aneuploid **^**cells**

**Chromosome**	**Abnormality**	**Frequency (out of 19 cells examined)**
Y	Gain	19
3	Loss	19
6	Loss	18
7	Loss	5
8	Loss	18
11	Loss	16
12	Gain	19
13	Loss	3
14	Loss	4
15	Loss	19
16	Loss	12
17	Loss	5
17	(q11.2q25)ins(17)(q25;q25q11.2)	19
18	Loss	12
19	Loss	19
22	Loss	11

Despite the abnormal karyotype, the iPSC-K3^aneuploid^ cells formed colonies that displayed a morphology that is characteristic of human pluripotent stem cells and immunocytochemistry revealed that the majority of cells within the colonies expressed the pluripotency marker OCT4 (Figure 1C). In addition, FACS analyses revealed that close to all of the cells in the culture expressed the pluripotent cell surface markers SSEA-4 (99%) and Tra-1-81 (96%), but not the fibroblast marker CD13 (Figure 1D).

### Differentiation of iPSC-K3^aneuploid^ cells to hepatocyte-like cells

**Figure 2:**
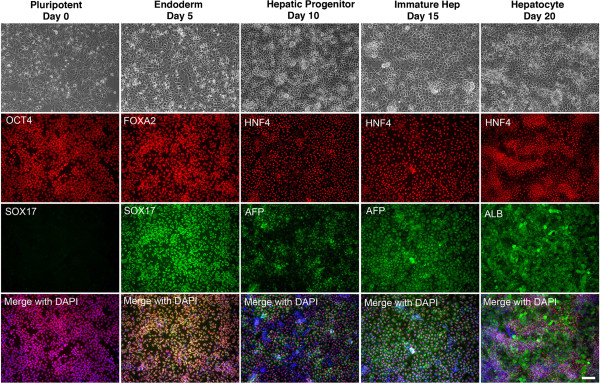
**Generation of hepatocyte–like cells from iPSC-K3**^**aneuploid **^**iPSCs.** The top row of panels shows phase contrast microscopy revealing the changes in morphology that accompany each stage of differentiation. Remaining panels document the result of immunocytochemistry performed on the cells to detect proteins that are characteristically expressed at specific stages of development. At day 0, prior to adding differentiation media or growth factors, the cells express OCT4, a marker of pluripotent cells, but do not express SOX17. At day 5, the cells have formed definitive endoderm, as seen by expression of the endodermal transcription factors FOXA2 and SOX17. At day 10, HNF4a, which is highly expressed in hepatic progenitor cells, can be identified in the majority of cells in the culture. At the same stage, low levels of AFP, a fetal hepatocyte marker, can be identified in a subset of cells. By day 15, almost all cells robustly express AFP indicating that they are forming immature hepatocytes. By the end of the protocol most cells express Albumin, which is highly expressed in adult hepatocytes. Scale bar = 100 μm.

To determine whether iPSCs harboring chromosomal variation could form hepatocyte–like cells in culture, we subjected iPSC-K3^aneuploid^ cells to culture conditions that we have previously demonstrated generates iPSC–derived cells with hepatocyte characteristics (http://www.stembook.org/node/721) [14]. The differentiation process was observed by following the expression of markers that reflect specific stages of hepatocyte differentiation by immunocytochemistry. Figure 2 shows that prior to the initiation of differentiation the cells expressed proteins associated with pluripotency including OCT4, whereas markers expressed in differentiated cells, such as SOX17 were not detected (Figure 2, Day 0). To generate definitive endoderm, Activin A, BMP4, and FGF2 were added to the culture during the first two days of differentiation, then Activin A alone was included for a subsequent 3 days. At this stage nearly all the cells expressed FOXA2 and SOX17, both of which are robustly expressed in the definitive endoderm (Figure 2, day 5). To generate hepatic progenitor cells, Activin A was removed from the medium and replaced with BMP4 and FGF2. After 5 days in culture nearly all of the cells expressed HNF4a, which is a transcription factor that is expressed in hepatic progenitor cells and is essential for their differentiation to hepatocytes (Figure 2, day 10) [15]. At this stage, some cells also began to express low levels of alpha-fetoprotein (AFP), which is another marker of the early hepatic lineage. After the addition of HGF for a further 5 days, which induces the hepatic progenitors to differentiate into fetal hepatocytes, the cells continued to express HNF4a and the cells increased expression of AFP, which became easily detected in nearly all of the cells in the culture (Figure 2, day 15). These fetal hepatocyte–like cells were then cultured for a further 5 days in Hepatocyte Culture Media supplemented with Oncostatin M. By completion of the protocol, nearly all of the cells expressed Albumin (Figure 2, day 20). During the differentiation process the morphology of the cells changed to eventually adopt a cuboidal appearance, with a high cytoplasmic to nuclear ratio, and evidence of glycogen granules and lipid vesicles observed in the cytoplasm (Figure 2). We confirmed that the differentiated cells retained an aneuploid phenotype by performing karyotype analyses after the completion of the differentiation protocol. As expected, the differentiated cells exhibited a low mitotic index, and very few cells were in metaphase at the time of cell harvest. A total of four metaphase cells were recovered; however, the chromosome morphology was poor and so we were unable to perform a thorough g-band analysis. Nevertheless, the chromosomal content of these cells was polyploid and the cells appeared to have a structural abnormality tentatively associated with chromosome 17.

**Figure 3:**
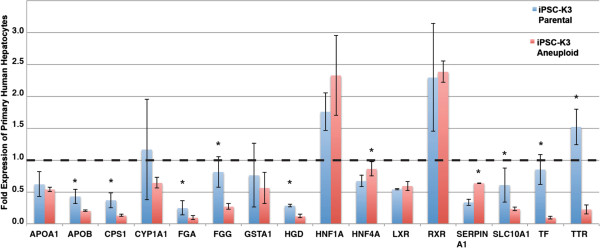
**Expression of hepatic mRNAs following differentiation iPSC-K3**^**aneuploid **^**cells.** Bar graph showing the relative levels of characteristic hepatic mRNAs identified by qRT-PCR in fresh human hepatocytes and in hepatocyte–like cells derived from iPSC-K3^aneuploid^ (red bars) and control iPSC-K3 (parental; blue bars) cells. The level of mRNAs detected in primary human hepatocytes was set to 1 (black dashed line). Specific mRNA levels in all other samples are presented relative to primary human hepatocytes. Error bars represent the standard deviation recorded from five (n = 5) independent differentiation experiments and p ≤ 0.05 was considered significant (*).

To gain a better understanding of the efficiency of differentiation we used qRT-PCR to determine the relative levels of several hepatic mRNAs in primary human hepatocytes and hepatocyte-like cells derived from either control iPSC-K3 or from iPSC-K3^aneuploid^ cells in independent differentiations (n = 5). As shown in Figure 3, the levels of *APOA1*, *APOB*, *CPS1*, *CYP1A1*, *FGA*, *FGG*, *GSTA1*, *HGD*, *HNF1A*, *HNF4A*, *LXR, RXR, SERPINA1, SLC10A1, TF,* and *TTR* mRNAs could be detected in all cell types. Although significant differences (p ≤ 0.05) in the levels of a subset of hepatic mRNAs were observed between the different lines, the hepatocyte–like cells derived from iPSC-K3^aneuploid^ cells exhibited an overall expression profile that was very similar to the parental cells. As we have described previously all iPSC–derived hepatocytes also retained expression of some fetal mRNAs including *AFP* and a number of mRNAs that are normally expressed in mature hepatocytes, including *CYP3A4*, were undetected in both control and iPSC-K3^aneuploid^ hepatocyte–like cells (data not shown).

### Hepatocyte–like cells derived from aneuploid iPSCs retain functional activities associated with primary hepatocytes

**Figure 4:**
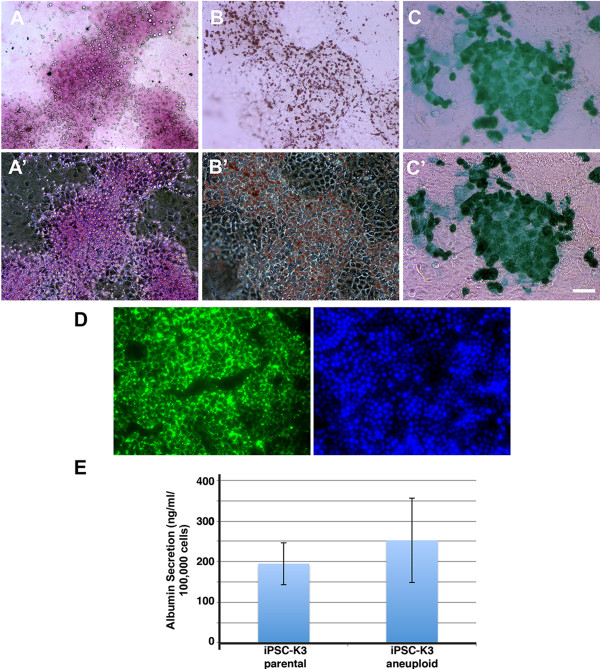
**Identification of basic hepatocyte functions in cells derived from iPSC-K3**^**aneuploid **^**cells.** Top panels show bright-field images with their corresponding phase contrast images below; scale bar = 100 μm. **(A, A’)** iPSC-K3 derived hepatocytes are capable of storing glycogen as shown by periodic acid-Schiff staining. **(B, B’)** Oil Red O staining demonstrates the cells’ ability to store lipids. **(C, C’)** Cells can take up indocyanine green. **D)** Differentiated cells were incubated with fluoresceinated low-density lipoprotein to demonstrate their ability to internalize LDL. The corresponding DAPI image is seen in the right panel. **E)** Bar graph showing levels, measured by ELISA, of human Albumin secreted into the culture medium from hepatocyte–like cells derived from either parental iPSC-K3 or iPSC-K3^aneuploid^ cells. Error bars represent the standard deviation recorded from three (n = 3) independent differentiations and no statistically significant difference in Albumin levels (p = 0.45) was observed.

The identification of proteins and mRNAs that are normally expressed during normal hepatocyte differentiation suggested that aneuploidy did not have a substantial impact on formation of hepatocytes from iPSCs. However, we recognized that this was a limited set of markers and so felt that it was important to determine whether the differentiated cells displayed activities that are normally associated with both primary hepatocytes and hepatocyte–like cells derived from euploid iPSCs [14,13]. The ability to store glycogen was assessed by Periodic Acid Schiff staining of cells (Figure 4A), oil red O staining revealed the presence of lipid droplets within the differentiated cells (Figure 4B), the cells were capable of the uptake of Indocyanine Green (Figure 4C), and incubation with fluorescently labeled low-density lipoprotein demonstrated the ability of the differentiated cells to uptake LDL (Figure 4D). Finally, we analyzed the supernatant in which the differentiated cells were cultured and observed that the iPSC-K3^aneuploid^ derived cells effeciently secreted Albumin (Figure 4E) at levels that were statistically indistinguishable from the iPSC-K3 derived cells. From these data, we conclude that the aneuploid status of the iPSC-K3 cells does not hinder their ability to differentiate into hepatocyte-like cells.

## Discussion

In the current study we have shown that cells with hepatocyte characteristics can be generated from iPSCs that harbor a severely abnormal chromosomal state. Although from a developmental perspective the successful directed differentiation of aneuploid iPSCs may seem surprising, the cells used in the current study retained representative loci from all chromosomes, albeit in an unbalanced state. It is likely that as regulatory networks are established during differentiation they stabilize through cross-regulation and so chromosomal imbalances may have little impact when cells are differentiated in culture. The iPSC-K3^aneuploid^ line also has a heterogeneous karyotype and the observed chromosomal abnormalities likely reflect the selective pressure of the culture environment. If a specific chromosomal arrangement were detrimental to cell viability or proliferation it would be lost during the culture of the pluripotent cells and if it were detrimental for hepatocyte formation it potentially could be selected against during the 20–day differentiation process.

Although in general it would be deemed prudent to ensure pluripotent stem cells retain a normal karyotype, it is possible that for some applications aneuploid cells could offer advantages. Indeed cancer cells with abnormal karyotypes have been used extensively for the study of cell function and activity because they can be easily grown in culture. Since iPSCs with abnormal karyotypes often grow robustly in culture they could be useful for applications that require large numbers of cells, such as drug screening and biochemical applications, especially if sub-lines can be identified that differentiate particularly well as a consequence of the chromosomal environment. In addition, comparative analyses between parental cells and aneuploid derivatives may be useful for the identification of loci that confer specific functional phenotypes, especially as they relate to cell growth. In the case of hepatocytes this may be particularly relevant given the variation in ploidy that is observed in adult hepatocytes and the potential contribution of ploidy to hepatocyte function.

## Conclusions

To conclude we have demonstrated that a human iPSC line that has extensive chromosomal abnormalities retains the ability to differentiate into cells that display hepatocyte characteristics. These data demonstrate that there exists considerable flexibility in the chromosomal content that is compatible with differentiation of pluripotent cells to the hepatocyte lineage.

## Methods

### Cell culture

Human iPSCs (iPSC-K3 [13]) were routinely cultured under low oxygen conditions (4% O_2_/5% CO_2_) in hES cell media (DMEM/F12 medium supplemented with 20% knockout serum replacement (Invitrogen), non essential amino acids (Invitrogen), glutamine (Invitrogen), penicillin/streptomycin (Invitrogen) and zbFGF (4 ng/ml)) on mitotically inactivated mouse embryonic fibroblasts (MEFs). Alternatively, cells were cultured on a recombinant E-cadherin-IgG Fc fusion protein matrix [16] (StemAdhere, Primorigen Biosciences, Inc) in MEF-conditioned hES medium or mTeSR [17]. When cultured on E-cadherin-IgG Fc, cells were passaged every 4–5 days by non-enzymatic methods using Versene/EDTA. All work carried out using human pluripotent stem cells was approved by the MCW Human Stem Cell Research Oversight Committee (hSCRO approval# 09–005) and all work performed using animals was approved by the MCW IACUC.

### Hepatocyte-like cell differentiation

Pluripotent cells were differentiated as discussed previously [14,18]. Briefly, pluripotent cells cultured on E-cadherin-IgG Fc were harvested using Accutase (Millipore) and plated onto 6-well tissue culture-treated plates pre-coated with 2 mg/ml Matrigel (Geltrex; Invitrogen). Typically, one 100 mm dish of cells cultured on E-cadherin-IgG Fc provided enough cells for 2 wells of a 6-well plate. Approximately 24 hours after seeding the cells onto Matrigel, when the cells were 85-95% confluent, differentiation was initiated by culture for 5 days with 50 ng/ml Activin A (R&D Systems) in RPMI/B27 (without Insulin) supplement (Invitrogen) under ambient oxygen/5%CO_2_. In addition, we included 10 ng/ml BMP4 (Peprotech) and 20 ng/ml FGF-2 (Invitrogen) for the first 2 days. This resulted in reproducible differentiation into definitive endoderm at efficiencies of greater than 80%. Cells were cultured for 5 days with 20 ng/ml BMP4 (Peprotech)/10 ng/ml FGF-2 (Invitrogen) in RPMI/B27 (containing Insulin) under 4%O_2_/5%CO_2_, then 5 days with 20 ng/ml HGF (Peprotech) in RPMI/B27 (containing Insulin) under 4%O_2_/5%CO_2_, and finally for 5 days with 20 ng/ml Oncostatin-M (R&D Systems) in Hepatocyte Culture Media (Lonza) supplemented with SingleQuots (without EGF) in ambient oxygen/5% CO_2_.

### Karyotype analysis

Pluripotent cells were cultured in T25 flasks coated with Matrigel in MEF-conditioned media for 2–3 days. Cells were transported to WiCell Research Institute (Madison, WI), who performed cell harvest and karyotype analysis of metaphase chromosomes using G-banding. For karyotype analysis of iPS cell-derived hepatocytes, cells were differentiated on 6-well tissue culture dishes coated with Matrigel. After three days of culture in Hepatocyte Culture Medium and Oncostatin M, cells were sent to WiCell Research Institute (Madision, WI) for cell harvest and analysis.

### Quantitative real-time PCR analysis

Total RNA was collected from cells at each stage of the differentiation using the RNeasy Mini Kit (Qiagen). Contaminating genomic DNA was removed using 1 μl of RNase-free DNaseI per 5 μg RNA. First strand cDNA was synthesized using MMLV-RT with dNTPs and random hexamer primers. Taqman-based qRT-PCR assays (PrimeTime) were obtained from IDT (Table 2) and PCR performed using an Applied Biosystems StepOnePlus™ Real-Time PCR System. All data were collected from reactions performed in triplicate.

**Table 2:** Primers used in real-time qRT-PCR assays

	**Probe**	**Primer 1**	**Primer 2**
**APOA1**	CTGCCAGAAATGCCGAGCCTG	CTTTGAGCACATCCACGTACA	GCCGTGCTCTTCCTGAC
**APOB**	CTGGATACCGTGTATGGAAACTGCTCC	CATTGCCCTTCCTCGTCTT	CCAGAGACAGAAGAAGCCAAG
**CPS1**	TCCAGCAATCATTCCGGCCAAGA	CCACAGGATTTAAGATACCCCAG	GTAATTGTTCAGCCACACCAAG
**CYP1A1**	TCTGTGATGTCCCGGATGTGGC	CCCAACCCTTCCCTGAATG	TTCTTCTCCTGACAGTGCTCAATC
**FGA**	CAGGCAGACGATCCTCATGGAAAACA	CAGCCCCACCCTTAGAAAAG	CTCCTTCAGCTAGAAAGTCACC
**FGG**	AATAAGGGAGCTAAACAGAGCGGGC	CAAAGACACGGTGCAAATCC	TTCCAGACCCATCGATTTCAC
**GSTA1**	CGGGCTGACATTCATCTGGTGGA	AAATCGCTACTTCCCTGCC	GGAAGCTGGAGATAAGACTGG
**HGD**	ATGCAGGCCACTCACAAAGTCTACTT	AGCAAGCCATTGTTAGACTT	CCTGATCCTAACCAGCTTAGATG
**HNF1A**	CAGGTTGGTGGTGTCGGTGATGA	GCCCTCTACAGCCACAAG	CAGTGTCTGAGGTGAAGACC
**HNF4A**	CAAGAAATGCTTCCGGGCTGGC	ATAGCTTGACCTTCGAGTGC	TGGACAAAGACAAGAGGAACC
**LXR**	TGCATAGCTCGTTCCCCAGCATTT	CCCTTCAGAACCCACAGAG	CGCAGCTCAGAACATTGTAGT
**RPL13A**	AGCAGTACCTGTTTAGCCACGATGG	GCCTTCACAGCGTACGA	CGAAGATGGCGGAGGTG
**RXR**	AGTACTGCCGCTACCAGAAGTGC	AGGACTGCCTGATTGACAAG	GACTCCACCTCATTCTCGTTC
**SERPINA1**	TGGTGCCTGAAGCTGAGGAGAC	AGCCAGGGAGACAGGGA	CTTAAATACGGACGAGGACAGG
**SLC10A1**	AACCTCAGCATTGTGATGACCACCT	TGTACAGGAGGAGAGGCATC	ACCTGTCCAATGTCTTCAGTC
**TF**	CTGTGTCAACTGTGTCCAGGGTGT	TGAGCCACGTAAACCTCTTG	AGTATTGGTTAAGGGTGGAGC
**TTR**	AGCAGCCTAGCTCAGGAGAAGTGA	CAGGTTTGCAGTCAGATTGG	CCATCCTGCCAAGAATGAGT

### Enzyme linked immunosorbent assay

Concentration of human albumin in cell culture supernatant was measured using the Human Albumin ELISA Quantitation Set (Bethyl; E80-129) according to the manufacturer’s instructions. Absorbance (OD) was read on a plate reader within 15 minutes at 450 nm. Raw values were converted to concentration based on the standard curve for each experimental run using ReaderFit (http://www.readerfit.com).

### Periodic acid Schiff staining

For glycogen detection, differentiated cells were fume-fixed by adding 1 ml PBS with calcium and magnesium to each well and 4% paraformaldehyde in the spaces between the wells. Fixation occurred by incubation at 37°C for 1 hour. Cells were permeabilized with 0.4% Triton X-100 in PBS for 20 minutes at room temperature. Control cells were incubated with Diastase (1 mg/ml in PBS; Sigma) for 1 hour 37°C. Cells were then incubated with Periodic acid (0.5 g dissolved in 100 ml nano-pure water) for 5 min at room temperature, washed with distilled water and incubated with fresh prepared Schiff’s reagent for 15 min.

### Low-density lipoprotein (LDL) uptake

LDL uptake assays were performed as previously described [19], based on published protocols [20,21]. At the end of the differentiation protocol, cells were washed 3 times with ice-cold PBS, then incubated in ice-cold Hepatocyte Culture Media containing 5 μg/ml BODIPY® FL LDL (Invitrogen) for 3.5 hours at 37°C. Unbound LDL was rinsed away with 5 mg/ml heparin in PBS before imaging.

### Indocyanine green uptake

Cellular uptake of indocyanine green (Cardiogreen; Sigma) was performed using indocyanine green at 1 mg/ml diluted in Hepatocyte Culture Medium (stock is 5 mg/ml reconstituted in water, stable for up to 8 hours). Differentiated cells were incubated for 1 hour at 37°C and rinsed three times with PBS before imaging.

### Oil red O staining

For the detection of lipid accumulation, differentiated cells were fixed with 4% paraformaldehyde in PBS for 20 minutes at room temperature, washed twice with nano-pure water for 5 minutes, then washed with 60% 2-propanol for 5 minutes. Cells were then incubated for 20 min at room temperature with a freshly prepared working solution. A 0.5% stock solution of Oil Red O was prepared by dissolving 0.05 g in 100 ml 2-propanol and incubating at room temperature overnight. The working solution contained 60% Oil Red O stock solution and 40% water, which was allowed to stand for 20 minutes at room temperature and then passed through a 0.20 μm filter before using. Cell nuclei were then counterstained with hematoxylin.

### Immunocytochemistry of cultured cells

Cultured cells were fixed with 4% paraformaldehyde for 20 min at room temperature, made permeable with 0.4% Triton X-100 in PBS for 15 min and blocked with 3% BSA in PBS for 1 hour. Cells were incubated overnight at 4°C with primary antibodies diluted in 1% BSA in PBS. Primary antibodies used were Oct3/4 (Santa Cruz; rabbit, 1:500), Sox17 (R&D Systems; goat, 1:250), FoxA2 (Novus Biologicals; Mouse, 1:1000), HNF4a (Santa Cruz; goat, 1:250), AFP (Sigma; mouse, 1:1000), and Albumin (DAKO; rabbit, 1:500). Primary antibodies were probed with respective secondary antibodies conjugated to Alexa Fluor 488 or 594 (Molecular Probes; 1:1000) and nuclei were visualized with DAPI.

### Flow cytometry analysis

Pluripotent iPS cells grown on E-cadherin-IgG Fc were removed from the plate by incubating with Accutase (Millipore) for 3 minutes at room temperature. Cells were blocked with 10% FBS (Gibco) in 1XPBS on ice for 15 minutes, then labeled with antibodies conjugated to fluorophores diluted 1:20 in 1% FBS (100 μl total) on ice for 20 minutes. Cells were washed with 1% FBS and resuspended in 1% FBS for analysis. Antibodies used were mouse anti-human CD13-488 (AbD Serotec), mouse anti-human SSEA-4-PE (Millipore FlowCellect), and mouse anti-human Tra-1-81-PE (Millipore FlowCellect). Unstained cells and cells stained with the appropriate fluorophore-conjugated isotype were used as controls.

## Abbreviations

iPSC: Induced pluripotent stem cell; ESC: Embryonic stem cell; FACS: Fluorescent activated cell sorting; LDL: Low density lipoprotein.

## Competing interests

The authors declare that they have no competing interests.

## Authors’ contributions

FKN: Contributed to experimental design, performed the majority of experiments and wrote draft of manuscript. MRD: Performed cell differentiations, qRT-PCR analyses, contributed to FACS studies and generated figures. JC: Contributed to qRT-PCR experiments. MAC: Performed LDL uptake assays. SKM: Performed albumin ELISAs. SAD: Contributed to experimental design, oversight of experiments performed, data interpretation, and wrote final version of manuscript. All authors read and approved the final manuscript.
